# Supplementary material for: Post-concussion symptom burden and dynamics: Insights from a digital health intervention and machine learning
Source: PLOS Digit Health. 2025 Jan 7;4(1):e0000697. doi: 10.1371/journal.pdig.0000697 (PMC11706386; doi:10.1371/journal.pdig.0000697)
Supplement: Attachment — Submitted filename: Response to reviewers.pdf [file pdig.0000697.s001.pdf]

Response to Reviewer comments

Dear Editor,

We would like to thank you and the reviewers for the comments and feedback. Please note that Reviewer 1 and Reviewer 2 had the same feedback, so we have responded it once under Reviewer 1. To help with the review process, we have put together a point-by-point response to address the reviewers' comments:

Reviewer comment	Response
**Journal requirements**	
1. Please send a completed 'Competing Interests' statement, including any COIs declared by your co-authors. If you have no competing interests to declare, please state "The authors have declared that no competing interests exist". Otherwise please declare all competing interests beginning with the statement "I have read the journal's policy and the authors of this manuscript have the following competing interests:"	I have read the journal's policy and the authors of this manuscript have the following competing interests:<br>Authors AJ, CD, DG and CH are shareholders in HeadOn Health Ltd which has an exclusive licence to commercialise the HeadOn intellectual property.<br>This statement has been added to the manuscript (page 2).
2. Please provide separate figure files in .tif or .eps format.	Included
**Reviewer 1**	
The development of the digital health tool is well described. Given that HeadOn is a web application, can the authors comment on the use of screens during this cognitive rest period post-concussion? How might this affect recovery? I note that a brief registration as well as Rivermead and PHQ are done in the initial phases. These are relatively short questionnaires, but has consideration been given to whether using a web application to complete clinical questions, document the symptom diary, and review educational materials on a daily basis soon after concussion increases cognitive overload and mental fatigue in these patients?<br>There is some evidence supporting the recommendation that patients abstain from screen time in the first 48-72 hours following a concussion to decrease time to symptom resolution, thus shortening the timeline to return to their usual daily activities.	Concussion guidance is to limit screen time early after an injury. A clinical trial looking at this question found that patients who abstained from screen time for the first 48 hours after their injury had a shorter recovery than those permitted to engage in screen time [1]. Although HeadOn is delivered mostly through a screen (mobile or desktop), there is the option of consuming some of the content through audio. Coupled with this, in the trial, the screen time–abstinent group had 130 minutes of screen time in the first 3 days after their injury. HeadOn can be delivered with limited screen time (5-10 minutes per day), which could easily be achieved within the 130 minutes of the intervention group quoted in the screen time RCT. Updated line 370.<br><br>1. Macnow T, Curran T, Tolliday C, Martin K, McCarthy M, Ayturk D, et al. Effect of screen time on recovery from concussion: a randomized clinical trial. JAMA Pediatr 2021 Nov 01;175(11):1124-1131 [FREE Full text] [CrossRef] [Medline]

Table 1: are the stages concurrent or consecutive	The stages are consecutive. This has been updated on Table 1 and a new column added to show the week numbers. Figure 2 has also been added to make the flow of the HeadOn program clearer.
Do you use “symptomology” and “symptomatology” synonymously?	All wording has been changed to symptomatology.
How was the 35 day cut off chosen for HeadOn intervention?	The decision around 35-day cut off was based on a number of factors. Firstly, we consulted individuals who had experienced a concussion during the co-development process. General opinion was that 35 days was an appropriate length of time for HeadOn to allow the necessary education and tasks to be delivered and completed. We also explored the literature to try and understand engagement levels and found evidence that individuals who had experienced a concussion were less likely to respond to EMAs as participants’ symptoms resolved (1). As the majority of patients recovery by around 1-month post-injury (2), we felt that a 35-day cut off was a reasonable and rational length for the HeadOn program. This has been added to the methods section (line 130).<br><br>*1- Sufrinko AM, Howie EK, Charek DB, Elbin RJ, Collins MW, Kontos AP. Mobile ecological momentary assessment of Postconcussion symptoms and recovery outcomes. J Head Trauma Rehabil. 2019;34(6):E40–8*<br>*2- Iverson GL, Gardner AJ, Terry DP, et al. Predictors of clinical recovery from concussion: a systematic review Br J Sports Med 2017;51:941–948.*
I am curious why the data analyst function of ChatGPT was used for analysis when Python can perform the modeling and visualizations.	The ChatGPT Data Analyst function was used because our team was not proficient with Python syntax, and the Data Analyst tool provided an accessible, user-friendly interface for conducting the necessary modelling and visualizations without requiring extensive coding knowledge.
2. Provide the rationale for selecting k means over other clustering algorithms.	K-means was chosen because it is a widely used and efficient algorithm for clustering when the primary goal is to group data based on proximity in a feature space, such as symptom severity in this study. The Elbow Method was used to select the optimal number of clusters, making it a suitable and straightforward method for clustering post-concussion symptom data, as stated in the discussion (line 327)

Provide some comment on the statistical power of the study sample for clustering analysis.	The study had 84 participants who provided a total of 758 symptom diaries, resulting in over 6000 individual symptom ratings. No formal power analysis was performed. However, clustering analysis typically requires large and diverse datasets to ensure the stability of the clusters. Given the significant differences in presentation characteristics and outcomes between clusters, we are confident that the study's power is adequate for this clustering analysis, despite the moderate number of participants.
How was missing data handled?	There was no missing data in the symptom diaries as all the symptom diaries were completed in full by patients. However, there was variation in the number of diaries completed per patient. The average number of diaries per patient was 9 over the 35 days of HeadOn. As stated in the methods (line 174), to address this variation, we averaged the symptom diaries for each patient to standardizes the data, for the K-means clustering.
In terms of the causes, I note that there is a question for whether this was a sports-related concussion (19%)- what were the main mechanisms?	Mechanisms were not recorded, users were given an option to select whether the mechanism was sport or alcohol related, but no further information was captured on the exact mechanism of the sports injury.
The range of days from injury to registration was about one week. What do you think is the effect of this variability on the severity of reported symptoms?	We added the following text to the manuscript (line 390) to answer this question: Alongside this, the fact that the average sign-up time to HeadOn was 7 days means that the data presented in the article represents a higher severity group who opted to access support later in their recovery journey.
Please provide some context for using Rivermead, PHQ-9, and FAST.	We opted to use Rivermead and PHQ-9 as they are validated questionnaires which have been well established in concussion research (1,2). We opted to use FAST as a it is a commonly used quick screening tool for alcohol misuse that can differentiate hazardous vs non-hazardous (3). This context has been added to methods section (line 120).<br>1. *Narducci DM, Moran B, Coris E, Tsalatsanis A, Graulich I, Del Rossi G. Predicting Concussion Recovery with PHQ-9 and GAD-7. South Med J. 2021 Dec;114(12):760-765. doi: 10.14423/SMJ.0000000000001336. PMID: 34853851.)*<br>2. *Maruta J, Lumba-Brown A, Ghajar J. Concussion Subtype Identification With the Rivermead Post-concussion Symptoms Questionnaire. Front Neurol.*

	2018 Dec 3;9:1034. doi: 10.3389/fneur.2018.01034. PMID: 30559709; PMCID: PMC6287109.)<br>3. THE FAST ALCOHOL SCREENING TEST Get access Arrow Ray Hodgson, Tina Alwyn, Bev John, Betsy Thom, Alyson Smith Alcohol and Alcoholism, Volume 37, Issue 1, January 2002, Pages 61–66, https://doi.org/10.1093/alcalc/37.1.61
Define "engagement rate" in Table 2.	Engagement rates with HeadOn tasks were defined as the percentage of participants who completed the particular task (eg: the percentage of participants who completed at least one thought diary during the HeadOn program). This has been clarified in the methods section (line 168).
Figures: I strongly encourage you to make the scientific figures accessible to readers with color-blindness. Provide a legend for abbreviations used in the radar plots.	Thank you. We have updated the figures to include annotations (including text or numbers) that allow clusters or bars to be identified clearly without relying on colour.
Discussion: the sentence "These clusters can be integrated into individuals' current electronic health record systems to assist in clinical decision-making" needs to be qualified. Is external validation using another dataset or further data from ongoing recruitment at your site required before using the clusters in clinical practice?	We have updated the manuscript with the following section (line 330): The symptom severity clusters we identified have the potential to support clinical decision-making and the delivery of targeted support to those in higher risk clusters. However, prior to translation into clinical practice, these clusters need to be both internally validated in a larger dataset from HeadOn (which is on-going) and undergo external validation from another concussion symptom dataset
**Reviewer 3**	
A bit more information about the HeadOn web-based tool would have been useful. This is likely a symptom diary with some educational resources. The daily diary is used to collect data on symptoms from the patients every day for 35 days. Does the HeadOn system require that the patient complete symptom diary at various points in time or just once a day (and at what time)? It is also not clear if the time at which the symptoms were entered were considered during the data analysis. The strength of ecological momentary assessment (EMA) technique is to access patient at multiple times and as they go by their daily activities (e.g., exercise, being tired, resting etc.), and it doesn't seem	As detailed in the methods section titled 'Digital Health Intervention', HeadOn is a structured digital intervention that combines educational, psychological and behavioural tools to support patients with their recovery. There is detailed information on the structure of the HeadOn program in Table 1 and also schematically in Figure 1 and 2. Completing the symptom diary is only one aspect of the HeadOn program. Participants are invited to complete the symptom diary once a day. A once-a-day approach was chosen based on two factors. Firstly, feedback during the co-design phase with patients showed that logging in many times a day would be overly onerous. The second reason was to minimise screen time for patients due to the potential negative impact on recovery. With regards to frequency of data capture, EMAs can be performed either multiple

that way. In order to inform personalized management for concussion patients we need better understanding of the dynamic fluctuations in the post-concussion symptoms within patients as well as between patients. Can the author comment on how symptom collection via HeadOn account for the diurnal variations and variations related to daily activities? In particular how lack of this information could have impacted the results? Also, whether once-a-day symptom diary could be truly regarded as EMA?	times per day or once a day. Once-a-day capture at a random time is well established and referred to as 'signal-contingent sampling'. The frequency of data capture depends on what you want to show. We were interested in subacute-chronic recovery periods after concussion, and single daily capture was an appropriate method to do this. A systematic review of EMA in TBI showed that studies vary from single to multiple daily data capture, showing our approach has been utilised in brain injury cohorts previously (1). We have added this detail in methods section (line 128) and in discussion (line 389)<br><br>1- *Mitchell, R.J., Goggins, R. & Lystad, R.P. Synthesis of evidence on the use of ecological momentary assessments to monitor health outcomes after traumatic injury: rapid systematic review. BMC Med Res Methodol 22, 119 (2022)*
One of the main symptoms that patients with concussion include Cognitive symptoms (e.g., difficulty concentrating and memory problems). Can the author comments how this was considered while designing headOn, given that it requires a lot of data entering and viewing of educational resources. There are many sensors and wearable, and voice activated approaches towards EMA. Why were they not used?	Concussion guidance is to limit screen time early after an injury. A clinical trial looking at this question found that patients who abstained from screen time for the first 48 hours after their injury had a shorter recovery than those permitted to engage in screen time (1). Although HeadOn is delivered mostly through a screen (mobile or desktop), there is the option of consuming some of the content through audio. Coupled with this, in the trial, the screen time–abstinent group had 130 minutes of screen time in the first 3 days after their injury. HeadOn can be delivered with limited screen time (5-10 minutes per day), which could easily be achieved within the 130 minutes of the intervention group quoted in the screen time RCT. Updated line 370**.** We did not include sensors in this stage of development, but this is an active area of research for us.<br><br>1. Macnow T, Curran T, Tolliday C, Martin K, McCarthy M, Ayturk D, et al. Effect of screen time on recovery from concussion: a randomized clinical trial. JAMA Pediatr 2021 Nov 01;175(11):1124-1131 [FREE Full text] [CrossRef] [Medline]
On line 123, "The weekly tasks vary by stage and compromise". I think you mean to say comprise.	This has been changed to comprise (line 125).
Regarding five stages of HeadOn:(1) Understanding your symptoms, (2)	The stages are consecutive. This has been updated on Table 1 and a new column added to

Sleep after a concussion, (3) Lifestyle and exercise, (4) Your thoughts, and (5) Getting back to baseline. Are these to be completed sequentially? The process is not clear. Perhaps authors might draw a diagram showing the dependency among the stages.	show the sequential weekly numbers. A new figure (Figure 2) has also been added to make the flow of the HeadOn program clearer.
The questionnaires Rivermead, PHQ-9, and FAST were used as baseline patient reported measures. Some rationale for their usage must be provided.	We opted to use Rivermead and PHQ-9 as they are validated questionnaires which have been well established in concussion research (1,2). We opted to use FAST as a it is a commonly quick screening tool for alcohol misuse that can differentiate hazardous vs non-hazardous (3). This context has been added to methods section (line 120).<br><br>1. *Narducci DM, Moran B, Coris E, Tsalatsanis A, Graulich I, Del Rossi G. Predicting Concussion Recovery with PHQ-9 and GAD-7. South Med J. 2021 Dec;114(12):760-765. doi: 10.14423/SMJ.0000000000001336. PMID: 34853851.)*<br>2. *Maruta J, Lumba-Brown A, Ghajar J. Concussion Subtype Identification With the Rivermead Post-concussion Symptoms Questionnaire. Front Neurol. 2018 Dec 3;9:1034. doi: 10.3389/fneur.2018.01034. PMID: 30559709; PMCID: PMC6287109.)*<br>3. *THE FAST ALCOHOL SCREENING TEST Get access Arrow Ray Hodgson, Tina Alwyn, Bev John, Betsy Thom, Alyson Smith Alcohol and Alcoholism, Volume 37, Issue 1, January 2002, Pages 61–66, https://doi.org/10.1093/alcalc/37.1.61*
Why a timeframe of 35 days chosen for the data collection? Is this the usual time by which the concussion symptoms eventually resolved in most patients? Perhaps some context with some evidence could be included.	The decision around 35-day cut off was based on a number of factors. Firstly, we consulted individuals who had experienced a concussion during the co-development process. General opinion was that 35 days was an appropriate length of time for HeadOn to allow the necessary education and tasks to be delivered and completed. We also explored the literature to try and understand engagement levels and found evidence that individuals who had experienced a concussion were less likely to respond to EMAs as participants' symptoms resolved (1). As the majority of patients recovery by around 1-month post-injury (2), we felt that a 35-day cut off was a reasonable and rational length for the HeadOn program. This has been added to the methods section (line 126).

	1- *Sufrinko AM, Howie EK, Charek DB, Elbin RJ, Collins MW, Kontos AP. Mobile ecological momentary assessment of Postconcussion symptoms and recovery outcomes. J Head Trauma Rehabil. 2019;34(6):E40–8*<br>2- *Iverson GL, Gardner AJ, Terry DP, et al. Predictors of clinical recovery from concussion: a systematic review Br J Sports Med 2017;51:941–948.*
The studied cohort registered for HeadOn an average of 7 days from injury. What was the impact of this time gap on symptom severity.	We added the following text to the manuscript (line 390) to answer this question: Alongside this, the fact that the average sign-up time to HeadOn was 7 days means that the data presented in the article represents a higher severity group who opted to access support later in their recovery.
"Data analysis and visualisation were conducted with Python using the Data Analyst function of ChatGPT: I am not clear what does this mean? Python was used for data analysis, so what was the purpose of Data Analyst function of ChatGPT?	The Data Analyst function of ChatGPT allows you to use Python for data analysis through natural language prompts. This was done as our team was not proficient with Python syntax, and the Data Analyst tool provided an accessible, user-friendly interface for conducting the necessary modelling and visualizations without requiring extensive coding knowledge.
Did the authors consider clustering algorithms other than k-means for clustering? If not, why not. What is the rationale for using k-means for this data? Are there any particular strengths of k-means with regards to this particular problem and the data set?	K-means was chosen due to its simplicity, efficiency, and suitability for partitioning data based on proximity in a feature space. This aligns with the goal of grouping patients by symptom severity. K-means performs well with moderate sized datasets making it appropriate given the size of the study. Its ability to handle continuous, numerical data, such as symptom ratings made it very well-suited to this dataset. Other analysis techniques considered were: Hierarchical clustering (computationally expensive and does not work well on noisy datasets) and Gaussian Mixture Models (allows clusters to overlap, which could capture symptom variations better but it assumes normal distribution within clusters, which does not hold here).
Some information on data preprocessing steps must be provided. E.g., how clean this data was, how was the missing or erroneous data handled. This data is self-reported, as well as time-series, were any specific methodological decision, e.g., data preprocessing strategies utilized with regards to these issues.	There was no missing data in the symptom diaries as all the symptom diaries were completed in full by patients. However, there was variation in the number of diaries completed per patient. The average number of diaries per patient was 9 over the 35 days of HeadOn. As stated in the methods (line 168), to address this variation, we averaged the symptom diaries for each patient to

	standardizes the data, for the K-means clustering.

We hope these comments and amendments meet your requirements. If you require any further information please do not hesitate to get in touch.

Aimun Jamjoom PhD FRCS(SN)
